# Mental health needs of the COVID-19 patients and staff in the Fangcang shelter hospital: a qualitative research in Wuhan, China

**DOI:** 10.1017/gmh.2021.23

**Published:** 2021-08-09

**Authors:** Jing Lu, Min Zhao, Qianying Wu, Chenyi Ma, Xiangdong Du, Xinchuan Lu, Qiufang Jia, Chuanwei Li

**Affiliations:** 1 Shanghai Mental Health Center, Shanghai Jiao Tong University School of Medicine, Shanghai, China; 2 Suzhou Guangji Hospital, Affiliated Guangji Hospital of Soochow University, Suzhou, Jiangsu, China; 3 Shanghai Key Laboratory of Psychotic Disorders, Shanghai, China

**Keywords:** COVID-19, Fangcang shelter hospital, medical workers, mental health, patients, qualitative research, Wuhan

## Abstract

**Background:**

During the coronavirus disease 2019 (COVID-19) pandemic, Fangcang shelter hospitals were opened in Wuhan, China, to isolate and care for patients with mild or moderate symptoms. The patients and staff in the hospitals faced mental health challenges. This paper reports the experiences and mental health needs from them.

**Method:**

Following the qualitative design, semi-structured interviews were conducted in the EastWest Lake Fangcang Shelter Hospital, Wuhan on March 2020. Data collection and analysis was based on grounded theory. Open coding was adapted and a structured codebook was developed through coding seminars. The themes and subthemes were then confirmed through thematic analysis. The findings were further explained and integrated in a theoretical framework.

**Results:**

A total of 10 COVID-19 patients and 13 staff, including doctors, nurses, psychiatrists, and policemen participated in the interviews. They have common needs, as well as their own needs. The perspectives from the staff also did complement for needs of the patients. The mental health needs were generalized into four themes, that is, basic needs, information and communication, emotional needs, and social support, each with several subthemes. In addition, there were some external factors that regulated the internal needs, which were summarized in a theoretical framework.

**Conclusions:**

The study indicates the directions on hospital management, mental health services, policy making, and social work to meet the mental health needs of the inpatients and staff from temporary shelter hospitals like Fangcang in Wuhan during the COVID-19 pandemic.

## Background

At the end of 2019, the new coronavirus disease 2019 (COVID-19) was discovered in Wuhan, China, and quickly evolved into a serious epidemic outbreak (Lu *et al*., 2020). After that, COVID-19 spread rapidly around the world, becoming a public health emergency. According to the World Health Organization (Organization), more than 61 million people worldwide have been infected with COVID-19 by 30 November 2020, and more than 1.4 million people have died.

As the epicenter of the COVID-19 pandemic in China, Wuhan was heavily burdened on the city's medical system. In order to solve the problem of insufficient beds in the designated hospitals and accelerate the admission of patients, many Fangcang shelter hospitals (FSHs) had been opened (Chen *et al*., 2020) since 5 February 2020 in Wuhan. FSHs were temporary medical facilities that converted from public venues, such as stadiums and exhibition halls, to isolate and care for patients with mild or moderate symptoms of COVID-19. A total of more than 12 000 confirmed COVID-19 patients had been treated in FSHs (Shu *et al*., 2020), which played a crucial role in the prevention and treatment of COVID-19 (Zhou *et al*., 2020; Wang *et al*., 2020*a*, 2020*b*).

COVID-19 had caused mental health impacts on both the patients and the medical health workers. The patients with confirmed or suspected COVID-19 experienced fear of the consequences of infection, and also boredom, loneliness, and anger (Xiang *et al*., 2020) in their quarantine. The frontline medical workers were also heavily pressured by risk of infection, inadequate protection, overwork, lack of contact with their families, and exhaustion (Kang *et al*., 2020*a*). A cross-sectional study (Kang *et al*., 2020*b*) of 994 medical workers in Wuhan found that about 63% of them had mental health problems.

Since the FSHs are greatly different from the designated hospitals on patient populations and environments, it is necessary to explore the mental health status of the patients and staff in FSHs. The previous quantitative studies (Dai *et al*., 2020; Gu *et al*., 2020; Li *et al*., 2020) focused on psychological symptoms of the participants, but did not include their experiences, feelings, needs, or the effects of some external factors. Therefore, in the study, mental health needs are defined as all self-reported thoughts and needs that may be related to mental health. Qualitative design is adopted to explore various mental health needs of the COVID-19 patients and the staff with different occupations in the FSH, so as to develop recommendations for the mental health service, FSH management, and policy making.

## Method

### Study design, setting, and participants

Qualitative research can improve the description and explanation of complex, real-world phenomena such as mental health needs (Bradley *et al*., 2007). In this study, semi-structured interviews were conducted and reported following the consolidated criteria for reporting qualitative research (COREQ) standards (Tong *et al*., 2007).

The face-to-face interviews were carried out in the EastWest Lake Fangcang Shelter Hospital. The interviewer (CWL), who worked in the hospital as a member of the Psychological Assistance Team from JiangSu, obtained permission for interviews from the management. The EastWest Lake Fangcang Shelter Hospital was one of the first three FSHs built in Wuhan and began to accept COVID-19 patients, mainly of mild symptoms, from 7 February 2020. There were 1440 beds, three wards, and several departments including medical, nursing, pharmacy, and radiology. The highest number of patients in the hospital was 1434 and the total number including all the staff was more than 3000 (Radio). The medical workers in the hospital were mainly from the assistance teams of Xinjiang, Ningxia, Guangdong, and Fujian.

The confirmed COVID-19 patients and the staff that aged more than 18 years and stayed more than 1 week in the hospital received our verbal invitation. And the individuals who responded to our invitation formed a convenience sample. They were asked to talk about their experience in the FSH after giving the informed consents. All the interviews took place in independent spaces.

### Data collection

The semi-structured interview outlines (Table 1) were completed and modified under the guidance of specialists in infectious diseases, psychiatry and psychology. We then conducted pre-interviews and make several adjustments to the problem descriptions.

**Table 1.** Semi-structured interview outlines

Participants	Questions
Patients	1. How is your life in the Fangcang shelter hospital?
	2. What is your inner feeling and thought recently?
	3. What problems are you facing and how to solve it? Do you need any help for that?
	4. How do the virus and the hospitalization affect you?
	5. What do you think about the future life after leaving the hospital?
Staff	1. What do you think about the patients and your relationship with them?
	2. How is your work in the Fangcang shelter hospital?
	3. What is your inner feeling and thought recently?
	4. What problems are you facing and how to solve it? Do you need any help for that?
	5. How does the emergency support work in Wuhan affects you?

According to arrangement of the hospital, the interviews were carried out on 7 March 2020, the day before the hospital was officially closed. The participants reconfirmed the informed consents, including their permission of recording and transcribing the interviews, and then filled out a short survey of demographic, physical, and mental information before the conversations.

Although the interviews were semi-structured, the method of grounded theory (Corbin and Strauss, 1990), a qualitative approach that could help finding out structural patterns from human experience, was fully applied in the process. According to grounded theory, all meaningful issues emerging in the previous interviews should be incorporated into the current one, and specific regularities and patterns should be found and verified. In every interview, we tried to validate previous findings and looked for new relevant factors. The conversations continued until the interviewer found no new meaningful viewpoints emerging.

A total of 19 interviews were completed, with 23 participants (10 patients and 13 staff). All the 10 patients and six staff were interviewed individually, and the rest seven staff were interviewed in 2–3 persons. They worked together in the hospital. In the interviews, they complemented and contrasted the answers of one another.

### Data analysis

The audio recordings were transcribed verbatim and checked for accuracy. All the thoughts, emotions, and demands of the participants in the 19 completed interviews were included in the data analysis.

The analysis was also based on grounded theory (Corbin and Strauss, 1990). Using the method of open coding, three researchers (JL, CWL, and QYW), including the interviewer, openly identified the data into concepts with practical meanings, and then compared, combined, and classified the concepts to generate codes. A structured codebook was developed as dataset of the inductive codes. In the coding seminars of the research team, the codebook was supplemented and modified until no new codes were proposed. The thematic analysis was then performed using the similar comparative analytic process used to produce codes, which was a key component of grounded theory. The generated themes and subthemes were reviewed and examined for rigor before making a list. We made connections to the themes, and all the connections were repeatedly verified, so that a theoretical framework was finally established.

The results of transcription and coding were checked and confirmed by the participants. No qualitative software was used.

## Results

The demographic characteristics of the 23 participants are shown in Table 2. The average age of the patients was 51.4 years. About 80% of them were women, and all of them were Wuhan natives. Meanwhile, the average age of the staff was 32.2 years, with different professions including nurse, doctor, psychiatrist, and policeman. About 38.5% of them were women, and only 7.7% of them were Wuhan natives.

**Table 2.** Demographic characteristics of the participants

Characteristics	Patient (*n* = 10)	Staff (*n* = 13)	Total (*n* = 23)
Mean age (range)	51.4 (32–65)	32.2 (21–50)	40.5 (21–65)
Gender			
Male	20% (2)	61.5% (8)	43.5% (10)
Female	80% (8)	38.5% (5)	56.5% (13)
Education			
Primary	30% (3)	0 (0)	13.0% (3)
Junior or High school	40% (4)	0 (0)	17.4% (4)
College	20% (2)	46.2% (6)	34.8% (8)
Bachelor or higher	10% (1)	53.8% (7)	34.8% (8)
Marriage			
Married	100% (10)	69.2% (9)	82.6% (19)
Unmarried	0 (0)	30.8% (4)	17.4% (4)
Occupation			
Worker	60% (6)	0 (0)	26.1% (6)
Retired	40% (4)	0 (0)	17.4% (4)
Nurse	0 (0)	53.8% (7)	30.4% (7)
Doctor	0 (0)	15.4% (2)	8.7% (2)
Psychiatrist	0 (0)	23.1% (3)	13.0 (3)
Policeman	0 (0)	7.7% (1)	4.3% (1)
Region			
Wuhan	100% (10)	7.7% (1)	47.8% (11)
Others	0 (0)	92.3% (12)	52.2% (12)

**Fig. 1.**
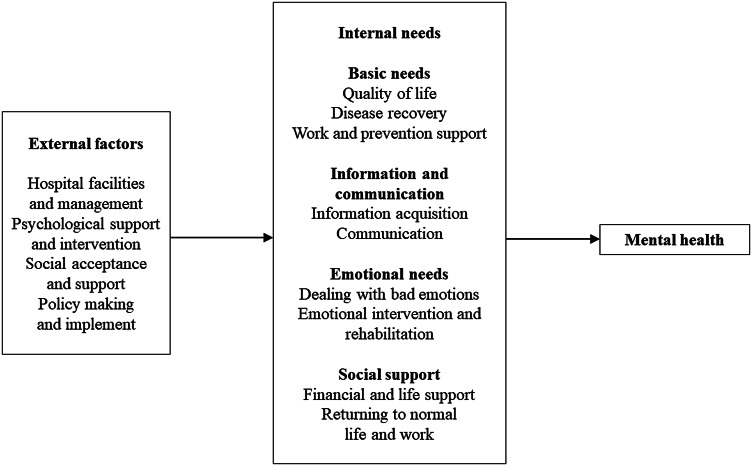
Theoretical framework.

The semi-structured interviews brought three perspectives, including self-reported needs of the patients and the staff, as well as the patient needs from the staff's perspectives. Exemplary quotes were used to illustrate the four themes and night subthemes (Table 3) that developed from the analysis. A thematic framework (Fig. 1) was established to summarize the themes and describe the relationship among them.

**Table 3.** Exemplary quotes supporting themes and subthemes

Theme and subtheme	Exemplary quotes
Patient needs	
Basic needs	
Quality of life	‘I did not have very good sleep at home, but at least I could sleep for five or six hours. However, I can only sleep for two or three hours here. I don't have enough energy during the day and I feel my body uncomfortable’. — Participant 7
Disease recovery	‘When I first came here, I was worried that cross-infection would happen between so many people…Now I have no symptoms or other health problems. My recent two nucleic acid tests have passed’. — Participant 4
Information and communication	
Information acquisition	‘We were transferred from section A to section B yesterday. No one came to us or talk to us, telling us where should we go today. I did not know that until I asked them myself today. When I was in section A, they always informed everyone timely, unlike here’. — Participant 8
Communication	‘Most of the time the communication with doctors and nurses is good. Sometimes I had too many questions to ask the doctor, which might trouble him and make him a little impatient. I used to complain about that, but now I can understand him’. — Participant 6
Emotional needs	
Dealing with bad emotions	‘I didn't know how I got infected. I'm old, over 60 years old, and I have diabetes and hypertension. To tell the truth, I had some negative thoughts about this disease. I felt there no way for me’. — Participant 2
Social support	
Financial and life support	‘If getting some life support, I will feel more comfortable. For example, when I came here, I brought the blanket myself. My families also brought blankets to their isolated places. After returning home, we will have no blankets to sleep on’. — Participant 6
Returning to normal life and work	‘I just want going to normal work after leaving the hospital. I worked in a landscaping enterprise and I'm not sure they would allow me returning to my used position’. — Participant 1
Patient needs from the staff's perspectives	
Emotional needs	
Emotional intervention and rehabilitation	‘The Fangcang hospital is a very suitable place for group activities of atmosphere mobilization and psychological rehabilitation. Positive guidance will be very helpful here. For example, our colleagues from the Xinjiang medical team organized folk dances, as reported on CCTV News’. — Group of Participants 20, 21, 22
Social support	
Financial and life support	‘Some of the patients have had five or six nucleic acid tests, each about three hundred yuan. They also had three or four CT imaging tests and other treatments…Many patients, especially those who felt their symptoms mild, might not be willing to come to the hospital for treatment at their own expense’. — Participant 19
Staff needs	
Basic needs	
Quality of life	‘Sometimes when I were off the night shifts and in the hotel bed, I couldn't fall asleep. I felt very stressed and thought over and over again whether there was somewhere unprotected…Many of our team, maybe one-third of the fifteen people, had taken sleeping pills, and some of them couldn't sleep without pills’. — Participant 19
Work and prevention support	‘I was afraid to put on and take off the protective gowns by myself at first. And then It got much easier that two people could do that together to help each other. Now it's no more a problem because there were specialized staff responsible for guiding and helping us’. — Participant 16
Information and communication	
Communication	‘We received doubts from one patient time and again. He said his nucleic acid tests had been negative before hospitalized, and it was the Fangcang shelter hospital that caused his recurrence. He repeatedly doubted the air, disinfection, etiology, and other professional things, and he never understood or accepted our explanations’. — Participant 19
Emotional needs	
Dealing with bad emotions	‘Actually, we were burdened and needed some help. For me, I usually felt uncomfortable and worried being infected in the free time. I felt uncomfortable all over my body’. — Participant 17
External factors	
Hospital facilities and management	‘The lights are too bright here at night, a major reason why many people can't fall asleep’. — Participant 4
Psychological support and intervention	‘The psychological assistance from your colleague helped me a lot’. — Participant 9
Social acceptance and support	‘Our community will help us on daily life’. — Participant 6
Policy making and implement	‘Sometimes I worried about the future and hoped that the policies would protect me’. — Participant 14

### Basic needs

This theme included the largest number of codes related to survival, health, and life.

‘I can only sleep for two or three hours here. I don't have enough energy during the day and I feel my body uncomfortable’. —Participant 7

Quality of life was a common need of the patients and staff. Due to the gathering people and facility conditions of the hospital, many patients had sleeping problems, including difficulty in falling asleep, reduced sleep duration, and irregular sleep pattern. The staff also reported sleeping problems. Many of them had taken sleeping pills for that, and some took the pills frequently or even every day. Another concern was the inadequate daily necessities in the hospital. Many materials had to be purchased outside, which usually took a long time. Besides, some patients, especially elderly ones, required some life care like helping them moving heavy things or finding someone.

‘When I first came here, I was worried that cross-infection would happen between so many people… Now I have no symptoms or other health problems’. — Participant 4

Disease recovery was another basic need for the patients. In addition to their current health, they also reported concerns on their transfer, quarantine, and subsequent treatment. Many patients had the worry that the cross-infection among people would affect their recovery, while those who did not meet the discharge criteria worried to be transferred to the designated hospital with severe pneumonia patients. Meanwhile, discharged patients would have to be quarantined in community for 14 days that might be too long for some of them. Other patients also focused on their subsequent treatments, recurrences, and sequelae.

‘I was afraid to put on and take off the protective gowns by myself at first… Now it's no more a problem because there are specialized staff responsible for guiding and helping us’. — Participant 16

Working in the FSH was a great challenge for all the staff, with work and prevention support one of their basic needs. One problem that troubled many staff was putting on and taking off protective gowns, so the hospital arranged responsible staff to guide and help them. They also reported that they were not so adapted to their work in the protective gowns at the beginning, as it was difficult to talk or perform medical operations. As to the current working hours, most staff thought them appropriate, while some of them worked heavily when the FSH was newly established.

### Information and communication

‘No one came to us or talked to us, telling us where we should go today. I did not know that until I asked them myself today’. — Participant 8

The interviewed patients thought it very necessary for them to obtain some important information in advance from the hospital, such as the arrangement of discharge or transfer, so that they had enough time to prepare. But sometimes the information might be sent quite late or even wrongly.

‘We received doubts from one patient time and again… He repeatedly doubted the air, disinfection, etiology, and other professional things, and he never understood or accepted our explanations’. — Participant 19

Communication was a critical element in hospitalization. Most of the patients had got enough communication, care, and assistance from the staff, while two of them thought that their doctor or nurse were sometimes impatient to them. For the staff, communicating with patients was part of their work, but sometimes they had to be pestered or doubted frequently by some of them, that took them a lot of time to deal with. Moreover, the rapid increase of patients and the lack of staff in the early stage of the hospital had caused difficulty in patient management.

### Emotional needs

This theme included all the emotions and related behaviors of the participants.

‘I didn't know how I got infected… To tell the truth, I had some negative thoughts about this disease. I felt there no way for me’. — Participant 2

Both the patients and staff had to deal with their bad emotions. As for the patients, the emotions included anxiety, depression, stigma, and worry. The first two were often described as ‘irritable’ or ‘hopeless’, and occurred in the early stage of infection. The stigma was the feeling of shameful or guilty for the disease, while the worry was mainly about their families, especially those who were also infected, and children or elderly people who lacked care. For the staff, anxiety, fear for infection, and stress from work were the most common negative emotions. These emotions were often hidden and only a few of them asked for help from their psychological or psychiatric colleagues.

‘Positive guidance will be very helpful here. For example, our colleagues from the Xinjiang medical team organized folk dances, as reported on CCTV News’. — Group of Participants 20, 21, 22

Emotional intervention and rehabilitation, on the other hand, were the attemption to improve positive emotions. From the experience of the interviewed staff, emotional outbreaks might happen on some relatively fragile patients, that could cause conflicts or even violence. They suggested that more attention and care should be paid on the emotional states of the patients, so as to detect those outbreaks from the beginning and prevent them from causing conflicts. Positive emotional promotion might also be effective for the psychological rehabilitation of the patients, especially those mild patients who got few medical treatments from the hospital.

### Social support

‘I worked in a landscaping enterprise and I'm not sure they would allow me returning to my used position’. — Participant 1

Returning to normal life and work was a great desire for the patients, although they thought the process not easy. Many of them were not optimistic that their employers and colleagues, communities, and neighbors would treat them in normal manners. They believed returning to work would be even harder than returning to community life for them, as the employers might make them long-term off work or even unemployment. They noted that relevant policies should be made and implemented to protect their rights of work and life, and also eliminate the discrimination.

‘When I came here, I brought the blanket myself. My families also brought blankets to their isolated places. After returning home, we will have no blankets to sleep on’. — Participant 6

The need of financial and life support was not mentioned by the interviewed patients but by the staff. They told us that the hospitalization was costly and many patients with mild symptoms would refuse to be hospitalized at their own expense, so the financial support from the government greatly improved the centralized isolation and prevention of the infectious disease. They also reported life support necessary for the patients, including the supplies, facilities, and logistics from the society.

### External factors

‘The lights are too bright here at night, a major reason why many people can't fall asleep’. — Participant 4

In addition to the above internal needs, we also found some external factors, including hospital facilities and management, psychological support and intervention, social acceptance and support, as well as policy making and implement. In a closed place for centralized isolation like the FSH, internal needs of the individuals were regulated by these external factors, and eventually affected their mental health. For example, many participants believed the too-bright light in the hospital night was a main reason of their sleeping problems. This defect in quality of life made effect on their mental health.

## Discussion

To our knowledge, this is the first qualitative study on mental health needs of the COVID-19 patients and the staff in FSHs during the pandemic of Wuhan. The present study reports the perspectives of the participants to fill the research gap of mental health needs. We also try to find objective requirements which are connected with many aspects of mental health work from the subjective results. These findings may provide a theoretical basis for other makeshift hospitals or temporary isolation places during COVID-19.

### Mental health needs

Basic needs of the patients and staff is one of our concerns. As temporary health facilities for the infectious disease, FSHs had complete infrastructure, adequate resource supply, systematic prevention, and management (Chen *et al*., 2020). Quality of life of the participants, however, involved some overlooked details, such as necessity purchase and life care. Life care service stations and staff can be very helpful for that. Similarly, medical information service stations can also be helpful, not only for patients to eliminate the anxiety of unknown, but also for medical staff to relieve some work burden. The FSH also placed emphasis on the prevention support for the staff, so that ‘zero infection’ had been achieved (Zhang *et al*., 2020*a*) during the whole medical activities in COVID-19. The long working hours, however, had caused job stress (Zhan *et al*., 2020) to the staff. More effort should be made by the hospital management to establish a complete work support system.

The emotional and psychological results are consistent with the previous researches (Bo *et al*., 2020; Zhu *et al*., 2020; Kang *et al*., 2020*a*; Li *et al*., 2020; Zhang *et al*., 2020*b*). Due to social isolation, perceived danger, physical discomfort, fear of cross-infection, and uncertain future, patients with COVID-19 often experience anxiety, depression, stigma, worry, insomnia, and posttraumatic stress symptoms, which cause harm to their social functioning and quality of life, and even lead to mental disorders (Rogers *et al*., 2020). For these emotional and psychological problems, Mukhtar proposed a Health Belief Model (Mukhtar, 2020) that emotions and health-related behaviors of individuals could be predicted and explained by their perceived stress, coping strategies, and some external factors. Therefore, it is necessary to improve the mental health work. Unlike general health facilities, FSHs also served as social space that provided necessary emotional support and social participation (Chen *et al*., 2020) to the patients. They supported one another and participated in social activities. For example, the EastWest Lake Fangcang Shelter Hospital where our interviews took place had been reported many times by the media (News) for the group XinJiang folk dance. Other activities included watching TV and reading (Daily C).

The relationship between the COVID-19 patients and the medical workers was seldom reported in previous studies, but we found that both of them thought the relationship important. The patients needed care, patience, and assistance from the staff, while the staff needed to manage a large number of patients, as well as deal with some doubts and conflicts.

In addition to the internal relationship, the social relationships outside the hospital were also great concerns for the patients. COVID-19 patients suffer severe stigmatization, especially in the workplace (Dang *et al*., 2020). Returning to their normal work and community life is difficult for them, so that policies should be made to protect their rights. Financial support, especially medical costs, was another important need of the patients, as well as the key that many individuals afford to receive treatment in the FSH. The medical bills of confirmed inpatients in China were RMB23 000 per person (China TSCIOOTPSRO), most of which were paid by the government.

The theoretical framework established from our findings indicated that the external factors regulated internal needs and thus affected mental health, which was consistent with the previous model. It is the first time, to our knowledge, to be proposed in the mild COVID-19 patients in centralized isolation. We suggest that making measures on the external factors can indirectly improve mental health of the patients and staff, which reflects the importance of promoting hospital management and related policies.

### Measures and recommendations

Some measures have been taken in the above aspects. The National Health Commission of China (China NHCOTPSRO) proposed that the confirmed COVID-19 patients and the frontline workers should be the major populations of psychological intervention. The Wuhan Municipal Government established psychological teams of professional psychologists and psychiatrists in the designated hospitals and FSHs, as well as online and telephone psychological service platforms (Liu *et al*., 2020). The WHO put forward measures (Organization) to protect the physical and mental health of medical workers. Some psychological intervention measures (China NHCOTPSRO; Wang *et al*., 2020*b*) had been adopted in the FSHs.

On the basis, and from the findings of this study as well, we make some specific recommendations to the psychological and psychiatric workers, hospital management, policy making, and social institutions. For temporary hospitals or isolation places in the pandemics, our recommendations would be helpful to promote mental health of the patients and staff.

For psychological and psychiatric workers, different levels of psychological support, intervention, and drug therapies should be provided to the patients and also the frontline workers according to their different psychological conditions. In the FSHs, various ways of support would be helpful, including education exhibition boards. group activities of rehabilitation, face-to-face consultation, online psychological service, psychological TV programs, etc. In order to prevent posttraumatic stress, long-term psychological follow-up, treatment, and rehabilitation should also be provided to the patients and staff in need.

For hospital management, hospital facilities and services should be improved, including life service centers that collect all perspectives on the hospital life and provide life service and care, information service centers that inform the patients of their conditions, discharge, and transfer arrangements, and provide inquiry service of laboratory and imaging reports, as well as communication facilities that maintain the communication of the individuals in the hospital with their families, friends, and the society outside. Management should also be improved, including a uniform, fair and efficient system of patient management that could relieve the burden of work on the staff, as well as a complete union system that provides prevention and protection to the staff, and ensure their appropriate working hours with breaks.

In policy making, the establishment and operation of FSHs or similar temporary facilities should be support. Current and follow-up medical expenses support should be provided for the patients, and extra support for those with difficulties. Polices should be made to regulate and supervise the hospitals, social institutions and communities to protect the rights of all patients and staff. And for social institutions, employers, and communities should help the patients returning to their normal work and community life, while mass media and social institutions should enhance publicity to eliminate the stigmatization of the patients and frontline workers.

### Limitations

The limitations of our work include that all the interviews were conducted by one interviewer. Our sample was also small. Future qualitative studies can be conducted in other countries and centralized isolation places to complement our findings. In addition, due to the qualitative method we use, this study cannot reflect the changes in mental health of the patients. Follow-up studies should be carried out in the future to find how the experiences in FSHs affect their long-term mental health. However, this study involves perspectives from the mild COVID-19 patients in centralized isolation and staff of different occupations. Our findings and recommendations are helpful to improve the management of makeshift hospitals or similar facilities so as to promote mental health of the patients and staff.

## Conclusions

In conclusion, the study has found mental health needs of the COVID-19 patients and staff in the FSHs. We have also made recommendations to psychological workers, hospital management, policy making, and social institutions, as the basis for promoting mental health of the patients and staff in makeshift hospitals during the pandemic.
