# Mental health needs of the COVID-19 patients and staff in the Fangcang shelter hospital: a qualitative research in Wuhan, China – ERRATUM

**DOI:** 10.1017/gmh.2022.8

**Published:** 2022-02-10

**Authors:** Jing Lu, Min Zhao, Qianying Wu, Chenyi Ma, Xiangdong Du, Xinchuan Lu, Qiufang Jia, Chuanwei Li

The publisher apologises that upon publication of this article the authors affiliations were not correctly assigned to the authors.

The correct listing is as below:

Jing Lu^1^, Min Zhao^1,3^, Qianying Wu^1^, Chenyi Ma^1^, Xiangdong Du^2^, Xinchuan Lu^2^, Qiufang Jia^2^, Chuanwei Li^2*^

1.Shanghai Mental Health Center, Shanghai Jiao Tong University School of Medicine, Shanghai, China

2.Suzhou Guangji Hospital, Affiliated Guangji Hospital of Soochow University, Suzhou, Jiangsu, China

3.Shanghai Key Laboratory of Psychotic Disorders, Shanghai, China

The online version of this article has been updated.
